# Noise-related Hearing Loss in Workers of a Thermal Reactor

**Published:** 2017-06

**Authors:** F.Ceyda AKINOCAL, Ramazan OCAL, Gulsun ADSIZ, Erol BELGIN

**Affiliations:** 1. Dept. of ENT, Gulhane Training and Research Hospital, Ankara, Turkey; 2. Dept. of ENT, Ankara Training and Research Hospital, Ankara, Turkey; 3. Dept. of Audiology, Konya Training and Research Hospital, Konya, Turkey; 4. Dept. of Audiology, School of Medicine, Medipol University, Istanbul, Turkey

## Dear Editor-in-Chief

Noise is the undesired, artificially developing voice of which quality and quantity is impaired. The most important objective effect of noise is on hearing. Noise-related hearing loss (NRHL) is one of the most common causes of hearing loss among adults. NRHL is a health problem known for long years and gained importance together with industrialization in Europe. The lower limit of noise that would lead to hearing loss is accepted as 85 dB SPL according to international standards (1). Hearing loss is not the only negative effects of noise; the others include psychological effects like behavioral disorders, nervousness, anxiety, and stress.

The aim of this study was to investigate noise-related hearing loss in the workers in a noisy workplace, to give educations about hearing loss, to take measures, to investigate the influences of noise on human psychology and prevalence of smoking.

This study was conducted in Beypazarı, Ankara, Turkey, in 2015–2016. For this study, the male workers in the same thermal reactor, which had 85 dB SPL or over noise level, were analyzed retrospectively. We evaluated the periodic follow-up results of pure tone audiometry and transient otoacoustic emission (t-OAE). The workers who had hearing loss due to other causes and chronic diseases requiring regular drug use were excluded from the study. Then we performed questionnaire forms to the workers. The results of 89 workers inclusion criteria were analyzed [mean age of 34.0 ± 8.7 yr (21–63 yr)]. The questionnaire form included questions about age, working years, daily working time, whether he received education about hearing protection, using protective equipment, presence of tinnitus and hearing loss, smoking status, anxiety, presence of dysphoria.

The protocol was approved by Ethics Committee of Turgut Ozal University (no: 99950669/72). SPSS ver. 17.0 (Chicago, IL, USA) was used for statistical evaluation. Friedman, Mcnemar, Chi-Square tests were used for analyses.

The injury caused by noise in outer hair cells leads to permanent hearing loss. These types of losses’ beginning slowly, symmetrically in both ears, from high-pitched frequencies (4000–6000 Hz), speech frequencies are affected later (2). In our study, 6 KHz was affected most in the right ear and 4 KHz was affected the most in the left ear, consistently with literature. T-OAE and “Distortion Product OAE (DPOAE) tests done in addition to audiologic examination is important for detection of the magnitude of inner ear injury and follow-up of recovery (3). In our study, hearing loss level and t-OAE results were not consistent.

NRHL is more during the first 10–15 yr of noise exposure. If exposure to noise is eliminated, the hearing loss threshold does not progress. An increase in abnormal audiogram frequency was observed as duration of noise exposure increases (4). In our study mean working years was found as 8.7±7.3 and hearing loss prevalence was seen to increase as working years increased (statistically significant *P*=0.04).

Smoking could be a risk factor for high-frequency hearing loss in a dose-dependent manner and potentiate the risk together with occupational noise exposure (5). We could not find a relationship between hearing loss and smoking in the workers; however, we consider that our results do not reflect the true, as we do not have data about the number of daily smoked cigarettes.

The influences of chronic industrial noise on psychological stress symptoms was analyzed [workplace satisfaction, discomfort after work, somatic complaints, anxiety and inability to enjoy life (depression)] and detected that noise increased these symptoms (6). We analyzed anxiety and inability to enjoy life and detected that these symptoms were statistically significantly higher among the subjects who had tinnitus. However, we could not find a relationship between these symptoms and hearing loss. These results show that tinnitus impairs quality of life in subjects, which should certainly be rehabilitated.

NRHL is a preventable condition and it does not have a specific treatment. Prevention of noise in its source and in the workers is the two approaches toward preserving hearing. Although prevention of noise in the worker is the last resort, education of the employer and the worker is very important. In our study, we inquired whether the workers received education about the relationship between noise and hearing loss before starting work and whether this influenced using ear protector and hearing loss. Forty-one (46.1%) workers had given education about hearing loss when starting work. Sixty-six (74.2%) workers were using ear protector. While 36 out of 41 workers (87.8%) who received education were using protective equipment, 30 out of 48 (62.5%) who did not receive education were using equipment. Ratio of using protective equipment was significantly higher among the workers who received education about hearing loss (*P*=0.007). A significant relationship was not found between using ear protector and hearing loss (*P*>0.05). This condition results from not using the ear protectors correctly and effectively, the workers’ preferring earplug, which limitedly reduces noise level instead.

It is important to support industrial audiology with more researches and to improve awareness of workers and employers in order to prevent noise-related Hearing Loss in the Workplace.

## References

[1] Rabinowitz PM (2012). The public health significance of noise-induced hearing loss. In: Le Prell CG Henderson D Fay RR Pooper AN, eds. Noise-Induced Hearing Loss Scientific Advances. New York: Springer, pp. 13–25.

[2] Halevi-Katz DN Yaakobi E Putter-Katz H (2015). Exposure to music and noise-induced hearing loss (NIHL) among professional pop/rock/jazz musicians. Noise Health, 17:158–64. 25913555 10.4103/1463-1741.155848 PMC4918652

[3] Prieve B Fitzgerald T. Otoacousic emissions, In: Kats J, editor. Handbook of clinical audiology. 6th ed, Baltimore: Wiliams and Wilkins; 2009; 497–512.

[4] Pelegrin AC Canuet L Rodríguez ÁA Morales MP (2015). Predictive factors of occupational noise-induced hearing loss in Spanish workers: A prospective study. Noise Health, 17:343–9. 26356377 10.4103/1463-1741.165064 PMC4900496

[5] Mizoue T Miyamoto T Shimizu T (2003). Combined effect of smoking and occupational exposure to noise on hearing loss in steel factory workers. Occup Environ Med, 60: 56–9. 12499458 10.1136/oem.60.1.56 PMC1740373

[6] Melamed S Luz J Green MS (1992). Noise exposure, noise annoyance and their relation to psychological distress, accident and sickness absence among blue-collar workers--the Cordis Study. Isr J Med Sci, 28:629–35. 1428822

